# Barriers to primary care among immigrants and refugees in Peterborough, Ontario: a qualitative study of provider perspectives

**DOI:** 10.1186/s12875-024-02453-x

**Published:** 2024-06-05

**Authors:** Madura Sundareswaran, Lucas Martignetti, Eva Purkey

**Affiliations:** 1 Department of Family Medicine, Queen’s University, Peterborough-Kawartha Site, 737 Victory Crescent, Peterborough, ON K9J 4T5 Canada; 2 Department of Family Medicine, Queen’s University, 220 Bagot Street, Kingston, ON K7L 3G2 Canada

**Keywords:** Immigrant and refugee health, Global health, Health systems, Health equity

## Abstract

**Background:**

Canada’s immigrants and refugees have often settled in large Canadian cities, but this is changing with rising costs of living and rural settlement initiatives. However, little consideration is made regarding systemic changes needed to accommodate this distribution, particularly in healthcare in medium-sized cities or smaller communities. For most Canadians, primary care is an entry point into the healthcare system but immigrants and refugees face unique barriers to accessing care compared to the general Canadian population. This project aimed to better understand the barriers to accessing primary care among newcomers in Peterborough, Ontario from the perspective of newcomer service providers.

**Methodology:**

Participants were recruited from community organizations identified by the local settlement agency, the New Canadians Centre, as having regular interactions with newcomer clients including clinics, not-for-profit organizations, and volunteer groups. Four focus groups were completed, each with three participants (*n*=12). A coding grid was deductively developed to guide thematic analysis by adapting Levesque et al.’s conceptual framework defining access to healthcare with five specific dimensions: approachability, acceptability, availability and accommodation, affordability, and appropriateness.

**Results:**

Participants identified lack of awareness of the healthcare system, stigma, competing priorities, and direct costs as some of the barriers for newcomers. Participants highlighted barriers unique to Peterborough including proximity to services, social isolation, and a shortage of family physicians. The results also highlighted strengths in the community such as its maternal-child health programming.

**Conclusion:**

The results provide a glimpse of the challenges to accessing primary care among newcomers in medium-sized communities and identify opportunities to prepare for changing settlement patterns.

**Supplementary Information:**

The online version contains supplementary material available at 10.1186/s12875-024-02453-x.

## Background

For most Canadians, primary care is an important point of entry into the healthcare system and encompasses a range of services including immunizations, preventative care, referrals to specialists, and mental health services [1]. Although access to primary care is an important determinant of overall health outcomes, there is a notable discrepancy in the accessibility of healthcare services across subsets of the population – particularly among immigrants and refugees [2, 3]. A commonly cited phenomenon known as “The Healthy Immigrant Effect'' has illustrated that the health of immigrants is often better than their Canadian counterparts upon arrival but later declines to or below that of the general population due to a complex range of socioeconomic, cultural, behavioural, and environmental factors [3–5]. Although this phenomenon only applies to immigrants – not refugees who arrive under less controlled circumstances – it sheds light on unique barriers faced by newcomers when accessing healthcare such as social isolation, language, transportation, or financial constraints which likely contribute to this health disparity [6].

Much of the existing Canadian literature on immigrant and refugee health is completed in large cities with populations greater than one million and higher rates of immigrant settlement such as Toronto or Montreal [7, 8]. However, as federal programs aim to draw more immigrants to small (population < 100,000) and medium-sized cities (population 100,000 – 1 million), newcomers are increasingly settling in smaller suburbs and rural regions [6, 9]. There is growing recognition that settlement programming, access to services, and the needs of newcomer Canadians vary between smaller communities and large urban centres [6, 10]. The settlement of newcomers outside of urban areas is not unique to Canada. In countries such as New Zealand, Australia, and the United States, more and more newcomers are moving into smaller communities to help fill labour shortages and offset population decline [6, 11]. Many newcomers also choose to settle in these areas because of greater employment opportunities and lower cost of living [6, 11].

Peterborough is a city in Southeastern Ontario, Canada, with a population of 83,000 [12, 13]. In Peterborough, immigrants represented just 8% of the population in 2016 [12, 13]. However, this pattern of settlement is rapidly changing. In the last five years, the number of immigrants who have moved to Peterborough has nearly doubled – from 625 between 2011-2015 to 1165 between 2016-2021 [13]. In 2016, the city also received official designation as a reception centre for government-assisted refugees in response to the Syrian crisis. Since then, with the help of the local settlement agency – The New Canadians Centre (NCC) – Peterborough has welcomed 765 government-assisted, and 190 privately sponsored refugees [14]. The resettlement target has nearly doubled since 2016 with 152 government-assisted refugees arriving to Peterborough in the last year alone, according to data from the NCC. However, little consideration is made to the systemic innovations, particularly in healthcare , needed to accommodate these changing patterns of migration [13].

### Service providers for newcomers

For many newcomers to Canada, the first points of contact upon arrival are non-profit, community-based settlement agencies that assist with systems navigation and community integration [15, 16]. In Peterborough this organization is the NCC, which works closely with local social and health-related organizations to meet the needs of newcomers across multiple sectors. In preparation for this project, the NCC helped identify community agencies that provided services to a high volume of newcomer clients which included medical clinics, volunteer organizations, schools, and not-for-profit organizations. Research on similar topics has been conducted using perspectives of service providers given their broader understanding of the structural and organizational influences on barriers to accessing care [15, 16]. As this was the first project of its kind to be conducted in Peterborough, researchers felt that exploring the views of service providers would be an appropriate initial step to understanding the landscape of experiences of newcomers in accessing primary care.

### Defining access to care

Although it is well known that access to care is important in evaluating and assessing a healthcare system, it has been difficult to define and measure objectively [17]. In recent years there have been several proposed adaptations to existing definitions of the term “access.”

In 2013, Levesque et al. created a conceptual framework of access to healthcare integrating many previous definitions. The final framework includes the characteristics of healthcare systems as well as the capacity of the people they aim to serve [17].

In this model, access to healthcare is defined as “the opportunity to identify healthcare needs, to seek healthcare services, to reach, to obtain or use healthcare services, and to actually have the need for services fulfilled” [17]. It consists of five specific dimensions: approachability, acceptability, availability and accommodation, affordability, and appropriateness [17].

Levesque’s framework is one of the most comprehensive to date. For experts in this field, it is gaining acceptance as it was developed with an extensive literature review and considers both health systems and the populations that they serve. It has been applied in qualitative research since its development and has been used in studying access to health for refugees worldwide [18–21]. For this reason, Levesque’s framework was used as a guide in both designing and conducting this study.

### Research question

Applying Levesque’s framework, this study aims to answer the following questions: 1. What are the challenges faced by immigrants and refugees in Peterborough, Ontario in accessing and utilizing primary care as perceived by newcomer service providers? 2. What changes can be made to existing services to improve access of primary care for immigrants and refugees? For the purposes of this study, primary care included preventative health, basic emergency services, referrals, mental healthcare , maternity care, and well child visits that may be provided by community health partners or organizations, in addition to access to a family physician or primary care nurse practitioner.

## Methodology

### Study design

This project was an exploratory qualitative study that aimed to evaluate barriers to accessing primary care from the perspective of service providers in health-related organizations and agencies caring for newcomers in Peterborough. Focus groups were the preferred method of data collection as they allowed participants to interact with each other, share and build upon each other’s experiences and give a more comprehensive view of the current landscape [22]. Although there is limited guidance regarding ideal sample size in qualitative research, a comprehensive review by Hennink et al. (2019) suggested that for a homogenous study population where focus groups are not stratified by demographic characteristics, 3-6 focus groups would be sufficient [23]. This is especially true to achieve “code saturation” which identifies the presence of issues in the data but may not provide a complete understanding of the issues or nuances, which instead refers to “meaning saturation” and may require a larger sample size [23]. The literature is sparse regarding the ideal number of participants in a focus group which can range anywhere from 1-20 participants [23, 24]. Some researchers advocate that the number of focus groups, not participants, should be the unit of analysis in conducting focus group-based studies [23, 24]. At the time of recruitment for this study, the goal was for each focus group to have approximately 5-6 participants to maximize opportunities for discussion.

The research team was prepared to adapt the study design in response to changing COVID-19 protocols to ensure that focus groups were conducted in compliance with the public health guidelines at the time. The literature was limited regarding the use of virtual focus groups for qualitative research prior to the COVID-19 pandemic. In virtual platforms, technological glitches, hesitancy to interrupt participants, reduced conversation, and more challenging interpretation of non-verbal cues can impact the flow of conversation [25]. Researchers decided that if focus groups were to be done virtually, it would be ideal to have no more than 3-5 participants per virtual room which has been supported by more recent research on virtual data collection in qualitative research [25, 26]. Ethics was obtained from the Queen’s University Health Sciences and Affiliated Teaching Hospitals Research Ethics Board prior to initiating research activities.

### Recruitment

To identify relevant stakeholders, researchers worked closely with the local settlement agency, the NCC, to understand what health-related services and supports were most often sought out by newcomers upon arrival. In designing this study, collaborators at the NCC highlighted the role that agencies across various sectors played in health systems navigation. Therefore, to obtain a more multidisciplinary and comprehensive view, the study included a broad range of agencies providing services to newcomer populations including health, educational, and social service organizations. A list was created containing relevant organizations and service providers for this study identified by the NCC. Participants were required to have had direct interactions with newly arrived immigrants and/or refugees within the city of Peterborough for at least one year in an employment or volunteer capacity. Participants also needed to have an active affiliation with an organization or agency serving newcomer populations thereby increasing the likelihood that they would be interacting with newcomers and assisting them in accessing the medical system at the time of the focus groups. By ensuring that participants were active in their roles at the time of the data collection, the researchers aimed to obtain the most relevant and up-to-date information regarding health system access.

Recruitment for this study took place during the COVID-19 pandemic. To maximize reach, invitations to participate in focus groups were distributed using digital posters. Potential participants and organizations were identified by the NCC and posters were emailed to individuals and administrators of relevant agencies. Recipients were encouraged to distribute to members of their team and other eligible parties to promote snowball sampling. Those who were interested were asked to contact the research team directly with an expression of interest to ensure that no supervisor-employee relationships were involved in recruitment. No honoraria were provided to participants.

### Data collection

Participants were invited to participate in focus group sessions. In accordance with COVID-19 public health protocols at the time, focus groups were conducted virtually via an online platform (Zoom). A total of four focus groups were completed, each with three participants and ranged from 48-88 minutes in length. Focus groups were facilitated by either MS or LM using a semi-structured focus group guide and conducted over two different days. The focus group guide is available in the supplemental material.

### Data analysis

The virtual focus groups were recorded and transcribed by professional transcriptionists. Due to the size of the community and challenges in preserving anonymity, the names of participants and the organizations that they represented were removed from the transcripts. A coding grid was deductively *developed (a priori)* using each of the dimensions of access as defined by Levesque et al. (2013) but it was agreed that new codes or subcodes could be developed and definitions modified during the data analysis process [17]. Existing literature on the dimensions of access were reviewed and incorporated in the final definitions used for the coding grid which are outlined Table 1. For example, Saurman et al. (2016) proposed that “awareness” be added as a dimension of access [27]. Although this was important, researchers felt there was significant overlap in Levesque et al.’s definition of “approachability” and ensured awareness was captured and made more explicit in the definition. Availability and accommodation were coded separately despite being a single item in Levesque et al.’s model since researchers felt that there may be sufficient data representing each.

**Table 1:** Definitions of Dimensions of Access (Adapted from Levesque et al., Penchansky et al., Saurman) [17, 27, 28]

**Dimension**	**Definition**
Approachability	Relating to the fact that people facing health needs can identify that some form of services exist, can be reached, and have an impact on the health of the individual. Services can make themselves more or less known among various social or geographical population groups. Various elements such as transparency, information regarding available treatments and services, and outreach activities could contribute to make the services more or less approachable. To capture Saurman’s addition to the original “dimensions of access” model, this definition was expanded to include awareness which states that understanding the local context and population needs can provide more appropriate and effective care. Patients can better access and use services if they were simply aware of them in the first place.
Acceptability	The relationship of clients’ attitudes about characteristics of providers to the actual characteristics, as well as to provider attitudes about acceptable personal characteristics of clients. Relates to cultural and social factors determining the possibility for people to accept the aspects of the service (e.g. the sex or social group of providers, the beliefs associated to system of medicine) and the judged appropriateness for the persons to seek care. It may be that some services are inequitable in the way they are organized, making them unacceptable to some sections of the community that they are intended to serve.
Availability	Given the number of codes identified in the initial analysis process, availability and accommodation were separated and distinguished by revisiting the original definitions for dimensions of access by Penchansky et al. (1981). Availability refers to the relationship of the volume and type of existing services (and resources) to the clients’ volume and types of needs. Availability constitutes the physical existence of health resources with sufficient capacity to produce services. It refers to the adequacy of the supply of physicians, dentists, and other providers; of facilities such as clinics and hospitals; and of specialized programs and services such as mental health and emergency care.
Accommodation	The relationship between how resources are organized to accept clients (including appointment systems, hours of operation, walk-in facilities, telephone services) and the clients’ ability to accommodate these factors. Also includes the relationship between the location of supply and the location of clients, taking account of client transportation resources and travel time, distance, and cost. It results from characteristics of facilities (e.g. density, concentration, distribution, building accessibility), of urban contexts (e.g. decentralisation, urban spread, and transportation system), and of individuals (e.g. duration and flexibility of working hours). It also relates to characteristics of providers (e.g. presence of health professionals, qualifications) and modes of provision of services (e.g. contact procedure and possibility of virtual consultations).
Affordability	The relationship of prices of services to the clients’ income, ability to pay, and existing health insurance. Client perception of worth relative to total cost is a concern here, as is clients’ knowledge of prices, total cost, and possible credit arrangements. It reflects the economic capacity for people to spend resources and time to use appropriate services. It results from direct prices of services and related expenses in addition to opportunity costs related to loss of income. It can vary by type of service and depends on the capacity to generate the resources to pay for care.
Appropriateness	The fit between services and clients’ need, its timeliness, the amount of care spent in assessing health problems and determining the correct treatment and the technical and interpersonal quality of services provided. It also will include adequacy of the services - including the appropriateness and quality of health services and its integrated and continuous nature. The definition was further broadened to include readiness or preparedness to use the services available.

Researchers MS and LM became familiar with the data set, reviewing all transcripts and developing the coding grid as above. Inductive codes were added during the analysis of Focus Group #1 and Focus Group #2 and the categorization and definitions of any new codes were discussed and agreed upon by researchers MS and LM. During the inductive coding process, the definition of appropriateness was expanded to include readiness or preparedness. A single document “code book” was developed to track any modifications to the code names and definitions in the early process and was also used to track comments by the researchers. In the data analysis of Focus Group #3 and #4, some sub-codes were reorganized or renamed to be more comprehensive or capture specific nuances, but no new sub-codes were added suggesting code saturation. A total of three rounds of coding were completed by authors MS and LM including cross-checking the data and discussions regarding any disagreements.

## Results

Each focus group had three participants for a total sample size of 12. Recognizing the challenges of preserving anonymity in a community the size of Peterborough, especially when working within the smaller niche of newcomer health, job titles and places of employment could not be published but will be described in broader categories. Participants represented a total of nine different organizations in Peterborough including three different non-profit or volunteer newcomer settlement organizations, five healthcare organizations or clinics, and one academic institution. Participants were varied in their occupations as well representing clinicians/healthcare workers (*n*=4), volunteers or employees of an organization serving newcomers (*n*=5), and administrators (*n*=3).

**Fig. 1:**
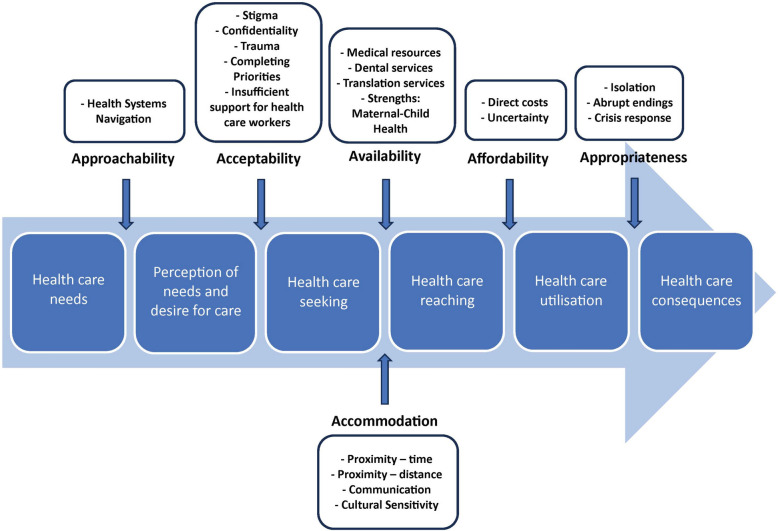
Barriers to Accessing Primary Care Among Immigrants and Refugees: Peterborough, ON

Once coded, the transcribed data were organized into six themes and are displayed in an adapted model of Leveque et al.’s framework (Fig. 1). The model below presents each of the five dimensions of accessibility of services. The elements within the arrow illustrate how access is realized ranging from perception of need to benefit from care. In representing it as a continuum, it demonstrates the importance of continuing care and stresses that access not only pertains to the first point of contact but is relevant each time a person tries to access a source of care [17].

### Approachability: challenges with health system navigation among newcomers and healthcare providers

Approachability is defined as knowledge and awareness that some form of health services exists. Participants shared that due to a lack of familiarity with the Canadian healthcare system, newcomers often struggle to identify what healthcare services exist and how to reach them. Participants also stated that newcomers may assume that the system operates in a similar fashion to that of their home country but quickly recognize this is not the case. In Canada, primary care is a critical entry point for access to specialists, wait times can be long, and simply knowing where and how to find primary care poses a significant challenge. Participants illustrated these unique barriers by sharing the experiences of their clients.

*“I would say the majority… [of newcomers] ... don’t understand the health system unless they have a family member here…[the] system is totally different than where they come from. For example, people will want to go and see a specialist first if they have been dealing with a cardiologist [where they come from], and then you say “no,” you have to be referred. You have to find a medical family doctor. And they question why…[I’d] been talking to a specialist before.” (P3, F1) *

*“{Family Doctors] with the Family Health Team all have after-hours clinics but to navigate that even for me is quite challenging…I have to call the office and then they tell me the number that I call to book an appointment for that particular day for the after-hours clinic. So it’s just like you have to go through three different phone calls in order to actually get to a person to book an after-hours appointment.” (P3, F2)*

But such knowledge gaps that influenced the approachability of healthcare services were not exclusive to the patients themselves. Participants discussed a lack of awareness of support for newcomers among healthcare providers as well. One barrier to care may simply be not optimizing existing services and infrastructure within the community.

*“The first thing that comes to mind is translation services, which unfortunately not everybody knows about…I just recently had a patient who didn’t speak English very well at our clinic, and there’s this sign that was posted…we have translation at the hospital. But there were already two other encounters prior to that one where the [staff] didn’t think to access the translation service. So the service is there, but it’s not always being accessed, unfortunately.” (P3, F2)*

*“I think the one thing is the mental healthcare access, which maybe is not clear because practitioners don’t know…what is the best or the most appropriate referral. I just found out that the Peterborough Youth Services provides mental healthcare up to age 25 for youth who are newcomers. I did not know that, and I’ve been here for, you know, a long time.” (P3, F4)*

### Acceptability: considering the impact *of stigma*, confidentiality, trauma, competing priorities, and expectations. For providers, insufficient support

Acceptability explores the relationships and attitudes of users and providers of the healthcare system towards each other. Evaluating the data allowed for the exploration of individual or systematic factors that influence access to care.

Participants identified six themes that influenced newcomers’ willingness to accept primary care in the existing system: stigma, confidentiality, trauma, competing priorities, expectations, and insufficient support for healthcare workers.

Many service providers discussed the influence of the stigma on care-seeking behaviours particularly around mental health. This included an unwillingness to discuss mental health, a lack of awareness that symptoms could be related to mental health, or not having the vocabulary to express mental health concerns.

*“So many newcomers might have undiagnosed mental issues when they arrive in Canada. Therefore, they don’t know that they need help.” (P3, F1)*

Closely linked to stigma, service providers discussed client concerns regarding the confidentiality of their health information. Not only are there issues with confidentiality by the simple nature of needing community interpretation services, but also being unaware of the rules of confidentiality between patients and their healthcare providers. Newcomers may be in precarious positions relating to housing or employment which may make them less likely to disclose information that they perceive could negatively affect them.

Participants shared different examples highlighting their experiences.

*“It’s a main barrier – language… you almost lose the confidentiality of the information…sometimes it’s difficult to even say [things] out loud when you have someone interpreting…from the community where you are going to see them tomorrow.” (P2, F4)*

*“Authority, for some of the newcomers, the authorities were problematic. The fact that they were authorities, what could the authorities do, that was a bit problematic in terms of approaching and their willingness to come…” (P1, F3)*

In contrast to the general population, newcomers’ willingness to seek care may be influenced by unique experiences of trauma. Not only was this discussed in the context of war but also with the migration process itself. As a result of many traumatic experiences including displacement, it was clear that there were some unmet health needs but also numerous competing priorities. Participants shared that secure housing and employment in Canada took precedence over seeking healthcare . If care was sought out, it was to address immediate physical needs rather than preventative or mental health concerns. Both of these themes are illustrated below.

*“I was going to say that the access to mental health is very much in terms of the timing as well. When newcomers are first here, if they have come through the refugee process they need that time to settle and make sure they have a roof over their head and food in their belly and a safe environment before they even consider that because they’ve been in crisis mode for X number of months, years sometimes. To deal with all the trauma of that they need to, first of all, be secure and set and then they need to see the value of dealing with that if they need to. I know with the one family, they didn’t want to go over all that. They just said, no, thank you. That was done. They’re here, that’s where they want to be. The trauma of dodging bullets and bombs and wild situations, they didn’t want to even go there. We didn’t end up trying to access services for [them] because they didn’t want to do that at the time. They talked about it a bit, but no.” (P1, F3)*

Patient expectations were noted to have an important role to play in the acceptance of services, particularly relating to the gender of the healthcare providers. Service providers shared examples of female newcomers requesting that their care be provided by female providers but there were also male patients who were not accustomed to female providers. Much of this was rooted in comfort, unfamiliarity, and anxiety – and participants highlighted examples of education to newcomer clients about accommodations that could be made, and many supported clients to prepare for these encounters which made for a more positive experience.

*“We had a couple of the women in the group were going to see the physician and they were pretty nervous about seeing a male physician at that point. We needed to make sure that there was somebody with them at the time, a female, and that they felt protected and secure at that point.*” (P1, F3)

Participants also highlighted the impact of insufficient support for healthcare providers to accommodate patients with complex needs including newcomers. For example, systemic barriers in the healthcare system such as compensation models that do not factor encounter times may impact clinicians’ willingness to accept newcomers to their family practices.

*“So again, I think a lot comes down to that time because if you’re using interpretation and you booked like a 20 minute appointment, 10 minutes is going to be in a language I don’t understand, 10 minutes is in a language I do understand. So you really, I feel, need to book longer appointments if there is interpretation or else we just squish it all in a smaller period of time. You don’t get paid for it. So systemic barriers.” (P3, F4)*

### Availability: limited medical, dental, and translation services; but an opportunity to learn from maternal-child health

Availability refers to the match between the volume and type of existing services to the needs of clients. It refers to the adequacy of the supply of physicians, dentists, and other providers; of facilities such as clinics and hospitals; and of specialized programs and services such as mental health and emergency care.

One of the most frequently discussed barriers to accessing primary care for both newcomers and Peterborough residents at large, was the shortage of local family physicians – with very few, if any, accepting new patients. Newcomers may be unable to access a family physician or nurse practitioner for months to years after arrival and must instead rely on episodic care through hospital emergency departments or walk-in clinics, which are also very limited in the region.

Participants talked about the challenges faced by this primary care provider shortage and discussed potential implications of this on the community.

*“Then the lack of family physicians in the community is a huge barrier and will increase the emergency department wait time and wait lists. That’s not a newcomer specific issue, that’s a community issue in Peterborough.” (P3, F3)*

*“And it’s a challenge in Peterborough because the access is so much more limited as compared to other places where most of them come from. You know, often you can walk in and see a doctor in their hometown within, you know, minutes of needing one.” (P1, F2)*

Participants shared frustrations regarding the absence of affordable dental services for newcomers which was identified as a high priority for this population. The availability of compassionate or subsidized dental care has been inconsistent over the years and at times based on the goodwill of individual dentists. Some participants alluded to the issues with such models most notably, sustainability.

*“We had compassionate dentists five years ago, say five, six, seven years ago. We run into one every once in a while and they would treat the person for very reduced cost. But then I think they, you know, we push them too hard…”(P2, F2)*

Participants identified translation to be a hurdle for many clients. Barriers relating to interpretation services included the cost, potential risk of loss of confidentiality, interpreter availability and coordination, and the challenges of translating medical vocabulary. Limitations in the availability of translated written documents were also mentioned. As some participants illustrated, working knowledge of a particular language may be insufficient to accurately address one’s health needs.

*“…initially, we were using a lot of the translation from New Canadians [Centre], which was excellent. One issue we found early on, though, was exactly what you mentioned. Some of them were not certified. Another thing I met with some early on was Syrian refugees, it’s a small community so a lot of them maybe are not comfortable. Even if they don’t know them, it’s still in their community…” (P1, F1)*

*“[Some] people have enough English that they can express themselves, so nobody feels obliged to access any of these telephone translation services. And that may or may not be a mistake, right? Because they may or may not be telling everything…or understanding the response from the professional, what’s recommended…but that’s part of their lives being frustrated over being able to communicate and understand the communications that’s, you know, the first five years people are here.” (P2, F2)*

Although this study was designed to explore barriers to accessing primary care, participants discussed the strength of maternal-child health programming and services in Peterborough, ON. Participants mentioned that pregnancy inherently presented an opportunity to enter the healthcare system but also discussed the availability of local clinics and programs geared to pregnant women and babies. The services and programs discussed would have been active at the time of data collection.

Those who are pregnant are better able to enter the healthcare system and receive prenatal and perinatal care from midwives, obstetricians, and the local family medicine-led obstetric service known as the Partners in Pregnancy Clinic (PIPC). Participants listed public health programs available in the community to better support new mothers and their babies such as the Healthy Babies, Healthy Children Program, and discussed programs that not only addressed the immediate medical needs of clients, but also basic needs including access to a food cupboard if needed. Care transitions were also better for these patients as the Pediatric Outpatient Clinic (POP Clinic) would often see babies and young children, and the Family Health Team had established a program trying to pair unattached babies with family physicians in the community. According to participants, pregnant patients represent a subset of the newcomer population that is able to enter the Canadian healthcare system more easily than their non-pregnant counterparts. The following quotations illustrate anecdotes shared by participants regarding helping clients navigate prenatal care in Peterborough.

*“if you are pregnant and having the baby that’s the best… you have at least two clinics who will take care of you and then the POP clinic will continue with your baby… you can have access to the doctor at the clinic and occasionally they might even transfer you…to one of the doctors in town. We don't see a major barrier in that regard when it comes to kids and pregnancy.” (P2, F4)*

*“I can’t imagine somebody pregnant - wherever they're coming from - is not going to seek care. And once they get in the system, whichever way they navigate through walk in, merge, self-refer, even public health. They won't be dropped, they’ll be at least directed to where to go.” (P1,F1)*

*“I have clients went to the Partners in Pregnancy and they got excellent service. And of course the health unit as well helping our clients with the vaccines, vaccinations and that’s really positive.” (P3, F1)*

*“The CPNP [Canada Prenatal Nutrition Program] program Babies First… they have a drop in lunch and they provide all sorts of food and health information…I think, goes up until they’re six months postpartum so they can bring their babies after. And there’s an LLC on site that will help with breastfeeding support, a lot of just different gift cards for like milk coupons and prenatal vitamins and all that kind of stuff… And then there’s like Healthy Babies Healthy Children is another really great program for public health that has like home visitors, nurses, home visitors that go into two women’s homes, either prenatally or postpartum. It’s a good program as well. Help with whatever baby needs and breastfeeding.” (P3, F2)*

### Accommodation: gaps in current infrastructure - time and distance, communication, and cultural sensitivity

Accommodation is defined as how well services are organized to accept clients. In discussing access to care for immigrants and refugees, time and distance were both identified as barriers. Limited hours of operations for routine healthcare services as well as increased appointment times due to interpretation posed unique challenges for newcomer patients. Participants also highlighted barriers to traveling to healthcare facilities within the city of Peterborough.

*“Accessing, there [are] great counselors here but I think still the time is limited and the hours and the sessions are limited as well. Interpretation would make that difficult.” (P2, F4)*

*“People have to miss school often, right? To go and access a service. They miss their own ESL or…their kids miss time from school…” (P2, F2)*

*"Are you going to explain to a newcomer how to get to the hospital, it involves transfers in multiple busses, and it's not in the most obvious area in town. It's not central.” (P1, F2)*

Participants discussed their experiences with communication barriers impacting access to health. This included language barriers and communication challenges extending beyond direct patient care. Booking an appointment over the phone, for example, was identified to be difficult with limited English vocabulary or being unable to use body language or hand gestures. Similarly, a lack of comfort and access to technology to facilitate communications were also mentioned.

*“Language is one big barrier and we often see that newcomer families, they’ll bring their children to their appointments because the children pick up the language a lot faster. You can wonder whether it is fair to the kids or whether you can trust the translation or not...” (P3, F3)*

*I would say just for in my role handouts as a barrier, we don’t have enough stuff that’s translated into different, you know, just culturally appropriate food handouts, things like that. But I always try to find things, but it’s not always easy to find things on every topic, especially in diabetes. (P3, F2)*

*“Quite often you need a phone to be able to make an appointment…but if there is a need to cancel or if there’s a need to reschedule or confirm appointment, they do not have a phone that they can give a phone number out to. If you’re waiting for tests they may not have a phone that they can receive calls to get test results and that sort of thing. Quite often the referral process is somebody will call you. So not having access to a phone or regularly having access to a phone, we experience an awful lot where that’s an issue.” (P2, F3)*

One participant shared that when it came to issues of mental health in Canada, for example, there is a expectation that individuals initiate communications regarding their unique health needs.

*“A kind of structural barrier that exists, too, is sort of the nature of seeking care in Canada. You know, Canadians are kind of assumed to self-advocate. You know, there’s an expectation that there’s a lot of education around like, you know, destigmatizing mental health issues, for example, and student and kids are getting this from a young age, which is great. But when someone shows up here in their twenties or thirties or older from a different context, culture, country, whatever, they may have different stigmas about seeking care. So there’s a lot of work to do in supporting newcomers to sort of break down those barriers and say it’s okay to acknowledge that you may need to talk to somebody about your mental health, or your physical health...” (P1, F2)*

Accommodation is also described as the connectedness between clients and providers. Participants shared their perspectives on how well the existing system accommodates a culturally diverse group of patients, suggesting that this is an area with potential for improvement.

*“Peterborough isn’t the most diverse place on earth. So I think that a lot of newcomers maybe don’t see themselves represented in the, in the people who are offering them care. And, you know, it’s an area that we should be mindful of and … improve in.” (P1, F2)*

*“I think every single service has a long way to go and there is always something more we could improve, no matter how sensitive they are. Our big learning is that there’s no way my staff will be able to memorize every single culture…So at the end I think we have to always ask and try to be open for any suggestions.” (P3, F3)*

*“We tried in some areas around food, for instance, because that’s one area we know is culturally different…we’re getting better at, you know, referring to a whole range of different foods. When we cook foods with groups, we try and cook culturally appropriate foods and use culturally appropriate examples. So we’re better, we're better at that I think.” (P2, F1)*

*“Even from my perspective as a care provider, I find we try and make people fit our system, we don’t change our system to try and best provide care for them.” (P3, F4)*

### Affordability: “How much?” - direct costs and uncertainty

Service providers working closely with newcomers discussed some of the direct expenses that may hinder or impede access to care. Many participants shared frustrations regarding the cost of receiving comprehensive dental services for this population. Despite access to the Ontario Health Insurance Plan (OHIP), uninsured health services such as prescription medications and medical devices (i.e.: glucose strips) can be costly and unaffordable for newcomers or as some participants shared, could come at the cost of something else. Such challenges, from the view of service providers, are shared below.

*“…so many newcomers are in precarious part time work, and they don't have access to dental or drug plans.” (P2, F1)*

*“…there is a specific medication that is not covered…and it is needed so the doctor would ask for it. But if you are in financial predicament then you are not able, which you want, pay the rent or get the medication? (P2, F4)*

In addition to the stress of the cost itself, there is an incredible amount of uncertainty among newcomers around whether they have healthcare coverage or insurance. For some, simply navigating supplemental insurance companies can be frustrating and time-consuming. In the event of an acute medical situation, this can result in uncertainty and stress regarding the final cost of a healthcare bill and not knowing what may or may not be covered. In addition, as newcomers experience transitions in their residency or academic status – such as from full-time to part-time students – they may or may not be aware of the implications to the healthcare coverage they are entitled to. Such uncertainty is also experienced by healthcare workers who may be unfamiliar with other insurance plans, but also may not accept alternative payment plans in their clinical settings. This can sometimes result in patients needing to pay upfront for services and later apply for reimbursement.

*“I would also say so that this particular patient that I recently was trying to help navigate without any kind of OHIP, UHIP or anything, and it took a long time. It took several weeks for her to finally get a clear answer on whether or not we could offer services…And it was me contacting a million people…trying to figure that out. And then the patient was just kind of like, am I going to get a bill at the end of all this?....It wasn’t straightforward to figure out how to make sure that there was going to be care that was funded.” (P3, F2)*

### Appropriateness: not a “one size fits all” approach - isolation, abrupt endings, and crisis response

Appropriateness can be best defined as the “fit” between the needs of patients and the ability to meet them. Having services and supports alone is not sufficient, they must also be adequate, and continuous. The definition was further expanded to include the concept of ‘readiness.’

Social isolation was common for newcomers and could influence their willingness and ability to seek care. The most frequently cited reason for isolation was the physical distance between newcomers and their support networks in their home countries. This can be slightly alleviated but not fully bridged by technology and messaging services. Cultural differences, such as Canada’s more individualistic societal structure were mentioned, as well as colder weather both being a shock for those from warmer climates and physically keeping people indoors and apart.

*“And I would just say like mental health services, if it's counseling for one person, but then their whole family is in another country. It only goes so far. I think isolation is a huge barrier and people just want their families nearby... “(P3, F2)*

For many newcomers, continuity of care was a huge problem. Newcomers frequently experienced abrupt endings to care at many steps in their healthcare-seeking journey. These abrupt transitions start even before newcomers enter the Canadian healthcare system.

*“Immunization, getting records was a barrier…That was because the newcomers had left in a hurry and didn’t have all their materials and couldn’t get it, couldn’t get access.” (P1, F3)*

However, participants also shared that despite being connected into the Canadian healthcare system, there continued to be a lack of continuity of care. For example, there were limitations on the number of counseling appointments available and the state of urgent care or walk-in clinics in Peterborough has been in flux over the years with clinics suddenly ceasing operations.

An important factor in discussing the adequacy of any programs for refugees or immigrants was the preparedness to accommodate newcomers to the region. Local programs to address the needs of newcomers were often reactive, adapting to crises as they occurred rather than being anticipatory or planned. Programs that were often developed ‘on the fly’ required quick mobilization and relied on community and volunteer support. This was demonstrated with a few examples such as organizing immunization clinics that could accommodate high volumes and language barriers when refugees from Syria had arrived in Peterborough in 2016.

*“...having a large number of Syrian refugees arrive really forced us to confront the language barrier…but we finally got our system sort of organized with some help from the New Canadians Centre to enroll and develop a whole policy and procedure around interpreters and primarily telephone because that is so much more accessible.” (P2, F1)*

## Discussion

This study addresses a gap in the literature by assessing barriers to accessing primary care among newcomers to medium-sized Canadian cities. These findings correspond to what has been reported in the Canadian literature to date where factors such as language barriers, lack of knowledge of the healthcare system, and competing priorities disproportionately affect newcomers’ access to health care [2, 29–31]. This study also sheds light on the compounded effect of being a newcomer and moving to a smaller community where access to healthcare services may be more limited in contrast to larger one – a concept that has been referred to as the “double burden of rural migration”[6].

### Medium-sized communities are unique

In 2020, authors Agrawal and Sangapala conducted a study addressing for the first time whether community size had an impact on the settlement process. The authors of this paper reported that research on refugee settlement experiences in small to medium-sized cities is lacking, and more work is needed in this area [32].

Medium-sized communities do not have single centralized hubs for newcomers to access all resources, knowledge, and support. In larger cities, gaps in the delivery of culturally and linguistically appropriate services are often filled by local denominational and non-denominational organizations, many of which are tailored to specific ethnic or cultural groups [32]. Such faith, ethnicity, or language-based groups do not exist in the same volume in small or medium-sized cities. As participants in this study discussed, access to healthcare for newcomers in Peterborough is fraught with fragmented institutional linkages and limited awareness of existing supports. Such findings highlight the role of community organizations in larger centres and suggest that perhaps more support is needed in small to medium-sized centres to fill the gap in health service delivery in other ways.

Barriers to primary care can be addressed through direct, intentional programming for health promotion and care for refugees. Refugee health centres, for example, often use a centralized, holistic model to connect newcomers to appropriate care and connect patients to other services such as financial assistance, social services, family and child welfare services, and cultural and religious organizations. This is often made possible through partnerships with community organizations [33–35]. Patients in these settings are given comprehensive care by providing services through a trauma-informed lens, establishing long-term, trusting relationships, and ensuring continuity of care [33–35]. These models can be effective in increasing access to care. McMurray et al. (2014) found that the introduction of a dedicated refugee healthcare clinic resulted in a 30% reduction in wait times to see a healthcare provider, 45% reduction in referrals to physician specialists, and nearly two-fold increase in connections to non-physician care providers such as therapists, dentists and optometrists [35]. In Lethbridge, a multidisciplinary healthcare clinic was established as a one-stop-shop for refugees who would access a family physician within 48 hours of arrival which covered oral health, immunizations, and labs for testing and screening [32]. This clinic was recognized by settlement agencies and private sponsors of refugees as a notable accomplishment. Other effective models include case management or system navigation programs with strong connections to local primary care providers, which can result in increased use of primary care, improved health outcomes related to mental health and preventative care, and increased client satisfaction following implementation [36–38].

The results of this study identified numerous barriers under each of the dimensions of access with participants illustrating how the size of the community may further impact confidentiality, service availability, distance between facilities, and isolation.

### Readiness over crisis response

The readiness of smaller communities to accommodate newcomers in existing primary care services is important as increasing numbers of countries are seeing migration outside of traditional gateway cities to fill labour shortages and offset population declines. However, many smaller communities lack sufficient physical infrastructure, social services, and ethno-cultural resources [6]. Most recently in Canada, the federal government implemented the Rural and Northern Immigration Pilot to spread the benefits of economic immigration to smaller communities. Immigration through this program offers a direct path to permanent residency for skilled workers only and although still in its infancy, programs such as this illustrate general interest in increasing diversity and shifting migration patterns that will directly impact smaller Canadian communities [39]. Furthermore in December 2023, Immigration Refugees and Citizenship Canada (IRCC) announced that over the next three years Canada hopes to welcome 51,615 refugees through the government-assisted refugees program. The IRCC currently lists 15 communities in Ontario as delivering resettlement services – which is the first point of contact for many refugee newcomers. Of these cities in Ontario, 1 has a population greater than 1 million; 3 have populations of 100,000-1 million; and 4 have populations of less than 100,000 [40]. Furthermore, IRCC is actively looking to expand and increase their services to other communities, many of which will be small to medium-sized [9]. It is a pivotal time in Canada to begin prioritizing equipping these communities for increased newcomer settlement instead of waiting for the next refugee crisis [40].

A common theme that emerged across several dimensions of access was crisis response and knee-jerk reactions to tackle issues faced by newcomers. Participants discussed a lack of planning and absence of plans or procedures for rapid settlement, as one would expect in a refugee crisis, so support and services needed to be quickly mobilized. Participants shared some initiatives that took place after the Syrian refugees arrived in 2016 but mentioned the challenges of sustaining programs when depending on the goodwill of others. This “compassion fatigue” may be more prominent in smaller communities as fewer people are involved for extended periods of time in resettlement activities [31].

Participants in the study shared that there may be opportunities to increase knowledge and awareness of cultural differences among providers in Peterborough. Building structured care models for newcomers and training staff to offer culturally sensitive and appropriate care is key, particularly to provide safe, considerate practices for newcomer groups such as refugees who may have experienced trauma [36, 41]. Ongoing cultural sensitivity training, education, and promotion of inclusivity although always important, may need to be more of a priority in less ethnically diverse communities [42].

### Lessons learned from maternal-child health

Although designed to evaluate barriers to accessing health care, this study identified Peterborough’s unique strengths within its healthcare system. Maternal-child health in Peterborough was recognized as a successful model for providing primary care to newcomers and addressing their needs. The nature of pregnancy mandating entry to the healthcare system and the inherent structure of appointments based on the progression of pregnancy lends itself to continuity of care. Participants shared examples of how maternal-child health services were provided by numerous organizations and sectors but remained effective at ensuring smooth transitions of care. In addition, participants outlined that service provision extended beyond direct clinical encounters but included assistance with meeting basic needs such as food or grocery gift cards. The emphasis on these services and some of their success may be attributed to a greater need for maternal-child health services among newcomers and subsequent higher use [38]. As a city, Peterborough appears to have been successful in primary care service provision within a subset of the newcomer population and some of these strengths could be used to model future programs and services.

### Limitations

This study was done from the perspective of service providers working with newcomers but did not interview refugees and immigrants in Peterborough who may provide unique insights from firsthand experience. Given the size of Peterborough and the relative proportion of newcomers in the community, the sample size of this study was small. The findings of this study are limited to what is communicated by newcomers to service providers through the course of their employment and volunteer work and may not reflect barriers to accessing primary care that may be sensitive or difficult to discuss in a focus group setting. To further explore barriers faced by newcomers accessing primary care in medium sized communities, future studies should consider incorporating the perspectives of newcomers themselves as well as larger sample sizes of all participants.

Future studies may also benefit from including participants from multiple medium-sized cities that have similar rates of migration and patterns to better include a wider range of perspectives and approaches.

## Conclusion

The findings of this study support the growing body of literature on the impact of community size on the newcomer settlement experience. Medium-sized communities likely require more support to effectively deliver primary care services to newcomers. More research and capacity building are needed to prepare for shifting migration patterns in the upcoming years.

### Supplementary Information

Supplementary Material 1.

## Data Availability

The data that support the findings of this study in the form of transcripts cannot be shared openly to protect participant identity. An annotated code book with additional examples of de-identified quotations, categorized by theme, can be made available from authors to interested parties upon reasonable request. Definitions used in the coding process (Table 1) along with the focus group guide are included in this published study.

## Abbreviations

IFHP: Interim Federal Health Program
IRCC: Immigration, Refugees, and Citizenship Canada
NCC: New Canadians Centre
OHIP: Ontario Health Insurance Plan
PIPC: Partners in Pregnancy Clinic
POP Clinic: Pediatric Outpatient Clinic
UHIP: University Health Insurance Plan
