# Supplementary material for: Barriers to primary care among immigrants and refugees in Peterborough, Ontario: a qualitative study of provider perspectives
Source: BMC Prim Care. 2024 Jun 5;25:199. doi: 10.1186/s12875-024-02453-x (PMC11151623; doi:10.1186/s12875-024-02453-x)
Supplement: Supplementary file 1 — Supplementary Material 1. [file 12875_2024_2453_MOESM1_ESM.docx]

**Barriers to Primary Care Among Immigrants and Refugees in Peterborough, Ontario: A Qualitative Study of Provider Perspectives**

**Focus Group Guide**

Hello everyone, my name’s <name> and I’m a <title> working with the Department of Family Medicine at Queen’s University and I will be leading the focus group today.

The purpose of this research is to explore your experiences as service providers working with immigrants and refugees to help us identify some of the unique challenges to accessing and using primary care services in Peterborough faced by your clients.

Studies have shown that the overall health of immigrants declines over time - one of the reasons identified for this are barriers to accessing health care. We would like to explore this further.

An important entry point in the health care system, is primary care. Now, to most people that may just mean their family doctor, but it includes a much broader range of services including emergency services, mental health, maternity care, walk in clinics, public health, and preventative health.

Most immigrants and refugees who arrive to Canada first receive support from a broad range of social support services, cultural groups, school boards, public health units, etc. These service providers have good relationships with immigrants and refugees and are often first point of contact. But there is not a lot of partnership or collaboration between the healthcare sector and these service providers who know their communities best. For this focus group, we will refer to immigrants and refugees under the broader term “newcomers.” If you are speaking in the specific context of a subgroup such as immigrants only, refugees, migrant workers, you may specify.

This focus group session will help improve our understanding of your firsthand experiences helping immigrants and refugees navigate our healthcare system within their first ten years of settlement.

This focus group session will take no more than one and a half hours. With your permission, it will be audio recorded. The recording will be used to ensure accuracy in the analysis of the data and will be destroyed once the project has been completed. Individual responses will remain confidential, and any quotes used will be anonymized at the time of analysis and/or writing.

To better understand your background and experience, we will be asking some questions that could be upsetting. We want to stress that every question is voluntary, so at any point, you may choose not to answer a question or to finish the discussion altogether. If you find you need help to deal with these emotions following the focus group session, we encourage you to talk to someone you trust, and/or contact the Talk Now Mental Health Clinic at the number we have provided on the letter of information.

Your participation is entirely at your discretion and you may stop your participation at any time.

Any questions before we begin?

Please take a moment to read and sign the consent form if you have not already done so.

I am going to turn on the audio-recorder now.

**Part 1: Introductions**

1. Tell me a bit about the organization you work with.
2. Tell me about your role in that organization.

**Part 2: Barriers to access**

Now we’re going to talk a bit about what barriers your clients might experience accessing health care services. You may find some overlap in your answers to the questions being asked, and that’s perfectly fine.

1. From your experience, what health care services are being sought out upon arrival? Where are these services being sought upon arrival?
2. Are potential and existing clients aware of healthcare services available locally?

**Probe:** If not, why not?

**Probe:** If so, how are they made aware?

1. Are healthcare services easy to access for newcomers?

**Probe:** Are there supports that exist in Peterborough to help immigrants or refugees navigate these health care services? If so, what are they?

**Probe:** In your experiences thus far, are there any structural, systemic, or even physical barriers that you have encountered?

**If no examples given, suggest:**

- - - If services are provided in-person, is there anything about the location, such as signage or steps necessary to enter the location that prevents or could prevent newcomers from accessing it?
    - If services are provided virtually, are there potential barriers to access for a newcomer to understand and access the services being offered? Can you tell me about these?
    - Do the hours services are offered work well for clients who are newcomers? Why or why not?
    - Are there any direct or indirect financial barriers? How do your clients address or manage these barriers?

1. Have you yourself identified areas where clients who are newcomers would benefit from expanded services and resources? What potential changes have you identified?
2. Primary care includes preventative health such as colon cancer screening, breast cancer screening, immunizations, etc. Are newcomers aware of these preventative health services? If so, are they able to access these preventative health services? Is the current delivery of preventative care services effective for newcomers? Why or why not?
3. Another aspect of primary care involves maternal-child health (i.e: delivering babies, well baby checks). Are newcomers aware of how this care is provided in Canada? Are they able to access this care? Is the current delivery of maternal-child care services effective for those who are newcomers? Why or why not?
4. Is the current delivery of mental health services effective for those who are newcomers? Why or why not?
5. From your perspective, what have been some of the challenges faced by newcomers when accessing their family physicians in Peterborough?

**Probe:** Is the organization of service delivery, including things like scheduling, connecting with, meeting with, and following up with clients, working for newcomers accessing your services? Are language barriers addressed in a satisfactory way for clients? Are language and translation services available in Peterborough, and if so what challenges are associated with their use?

1. Do you feel that current healthcare services are culturally sensitive to the concerns of clients who are newcomers? If so, can you give me an example? If not, can you give me an example?

1. What frustrations have your clients who are newcomers expressed with regards to accessing health care services in Peterborough? Have you observed clients or those supporting them asking for health care services our community does not provide? If so, what services do they tend to ask for?
2. What positive experiences have your clients had accessing health care services in Peterborough?
3. Are there any other areas where immigrants in Peterborough may face barriers to primary care that we haven’t spoken about so far? Do you find that the services and resources available meet the needs of newcomers?

**Probe:** Have you observed clients or those supporting them asking for health care services our community does not provide? If so, what services do they tend to ask for?

**END**
